# Relationships between epicardial adipose tissue thickness and adipo-fibrokine indicator profiles post-myocardial infarction

**DOI:** 10.1186/s12933-018-0679-y

**Published:** 2018-03-16

**Authors:** Olga Gruzdeva, Evgenya Uchasova, Yulia Dyleva, Daria Borodkina, Olga Akbasheva, Ekaterina Belik, Viktoria Karetnikova, Natalia Brel, Alexander Kokov, Vasiliy Kashtalap, Olga Barbarash

**Affiliations:** 1 Federal State Budgetary Institution, Research Institute for Complex Issues of Cardiovascular Diseases, Kemerovo, Russia; 2 Federal State Budget Educational Institution of Higher Education, Kemerovo State Medical University of the Ministry of Healthcare of the Russian Federation, Kemerovo, Russia; 3 Autonomous Public Healthcare Institution of the Kemerovo Region, Kemerovo Regional Clinical Hospital named after S.V. Beliyaev, Regional Center for Diabetes, Kemerovo, Russia; 4 Federal State Budget Educational Institution of Higher Education, Siberian State Medical University of the Ministry of Healthcare of the Russian Federation, Tomsk, Russia

**Keywords:** Visceral obesity, Epicardial adipose tissue, Cardiac fibrosis, Adiponectin, Stimulating growth factor 2, Interleukin-33

## Abstract

**Background:**

Determination of the impact of visceral obesity and epicardial adipose tissue thickness on stimulating growth factor levels during hospitalization for myocardial infarction is of potential importance for predicting outcomes and assessing the development of cardiofibrotic changes associated with maladaptive myocardial remodeling. In this study, we aimed to investigate the relationships between epicardial adipose tissue thickness, adipokine profiles, and the stimulating growth factor 2/interleukin-33 signaling system during hospitalization for myocardial infarction, and with the cardiac fibrosis extent 1-year post-MI in patients with visceral obesity.

**Methods:**

Eighty-eight patients with myocardial infarction were grouped based on their visceral obesity. Serum leptin, adiponectin, stimulating growth factor 2, and interleukin-33 levels were measured on days 1 and 12 and at 1 year. The epicardial adipose tissue widths and the cardiac fibrosis areas were measured on day 12 and at 1 year.

**Results:**

Visceral obesity was associated with epicardial adipose tissue thickness increases, adipokine imbalances, elevated leptin levels, and lower adiponectin levels during early hospitalization, and cardiac fibrosis development. Patients without visceral obesity had higher interleukin-33 and stimulating growth factor 2 levels during early hospitalization and lower cardiac fibrosis rates. Epicardial adipose tissue thickness was positively associated with cardiac fibrosis prevalence and interleukin-33 levels and negatively associated with stimulating growth factor 2 levels. The cardiac fibrosis extent was negatively associated with interleukin-33 levels and positively associated with stimulating growth factor 2 levels.

**Conclusions:**

Increases in epicardial adipose tissue thickness are associated with cardiac fibrosis development 1-year post-myocardial infarction and are higher in patients with visceral obesity. The metabolic activity of the epicardial adipose tissue is associated with elevated interleukin-33 and reduced stimulating growth factor 2 levels.

## Background

Adipose tissue, particularly its visceral component, is central to the initiation of many pathophysiological processes, including cardiac fibrosis development [1]. The inflammatory response after ischemia–reperfusion injuries in infarct zones is accompanied by fibroblast to myofibroblast transitions and their migration to pathogenic scar sites. Early remodeling comprises stretching of the infarcted segment, ventricular dilation, left ventricular spherical remodeling, and collagen synthesis by the myofibroblasts [2]. Cardiac homeostasis during ischemia–reperfusion injury is supported by a network of direct and indirect interactions between the cardiomyocytes and resident cells, including fibroblasts, adipocytes, and endothelial and immune cells. The secretion of several inflammatory cytokines from epicardial adipose tissue (EAT) is accompanied by a reduction in the expression of adiponectin that has anti-inflammatory and anti-atherogenic effects in patients with myocardial infarction (MI) [3]. EAT increases and the initial elevations of proinflammatory markers and neurohumoral activity indices in the visceral adipose tissue (VAT) affect myocardial revascularization outcomes, resulting in complications [4]. The involvement of VAT in inflammatory responses renders it a key player in cardiac fibrosis development. The close proximity between atrial myocardium and EAT suggests that EAT-secreted adipokines can contribute to structural remodeling of the atrial myocardium, including modification of fibrosis. Indeed, conditioned medium from epicardial adipocytes induces fibrosis in atrial myocardium and promotes the transition from fibroblast into myofibroblasts [5, 6]. EAT seems to play an important role in the inflammatory process within the atherosclerotic plaque which contributes to progression of coronary artery disease [7, 8].

Stimulating growth factor 2 (ST2) is the most promising marker of early myocardial remodeling. It is expressed on the cardiomyocytes in response to biomechanical stress [9]. ST2 is a member of the interleukin (IL)-1 receptor family. ST2, which potentiates the effects of IL-33, mainly exerts anti-hypertrophic and anti-fibrosing effects on cardiomyocytes undergoing biomechanical stretching [10]. However, acute increases in the ST2 levels are accompanied by inhibitions of the favorable antihypertrophic effects of IL-33 and more intense development of cardiac fibrosis following myocardial damage [9, 11]. Investigating the impacts of visceral obesity and EAT thickness on the ST2 levels during hospitalization for MI may be important for predicting outcomes and assessing the development of cardiosclerotic changes associated with maladaptive myocardial remodeling.

This study aimed to assess the relationships between EAT thickness, the adipokine profiles, and the ST2/IL-33 signaling system during hospitalization for MI, and the degree of cardiac fibrosis at 1-year post-MI in patients with visceral obesity.

## Methods

The study was performed at the Federal State Budgetary Institution’s Research Institute for Complex Issues of Cardiovascular Diseases. The study’s protocol was approved by the local ethics committee. Patients were included in the study after they provided written informed consent.

### Study participants

Eighty-eight patients with MIs, comprising 65 men and 23 women, were included in the study. The patients’ mean age was 58 (range: 52.0–63.5) years. An MI diagnosis was verified using the diagnostic criteria from the Russian Society of Cardiology (2007) and those from the European Society of Cardiology, American College of Cardiology Foundation, American Heart Association, and the World Heart Federation Task Force for the Redefinition of Myocardial Infarction [12]. The control group comprised 30 healthy subjects without cardiovascular disease (CVD) whose mean age was 58.42 (range: 52.2–71.1) years. The inclusion criteria were the presence of characteristic chest pain that lasted > 15 min, electrocardiogram (ECG) changes, namely, ST-segment elevations in at least two consecutive leads, and laboratory test results indicating elevated creatine kinase (CK), CK-MB, and troponin T levels. The exclusion criteria were being aged < 50 or > 80 years, and the presence of concomitant diseases, including cancer, infections, mental disorders, chronic obstructive pulmonary disease, connective tissue diseases, and renal and hepatocellular insufficiencies.

### Imaging assessments

**Fig. 1:**
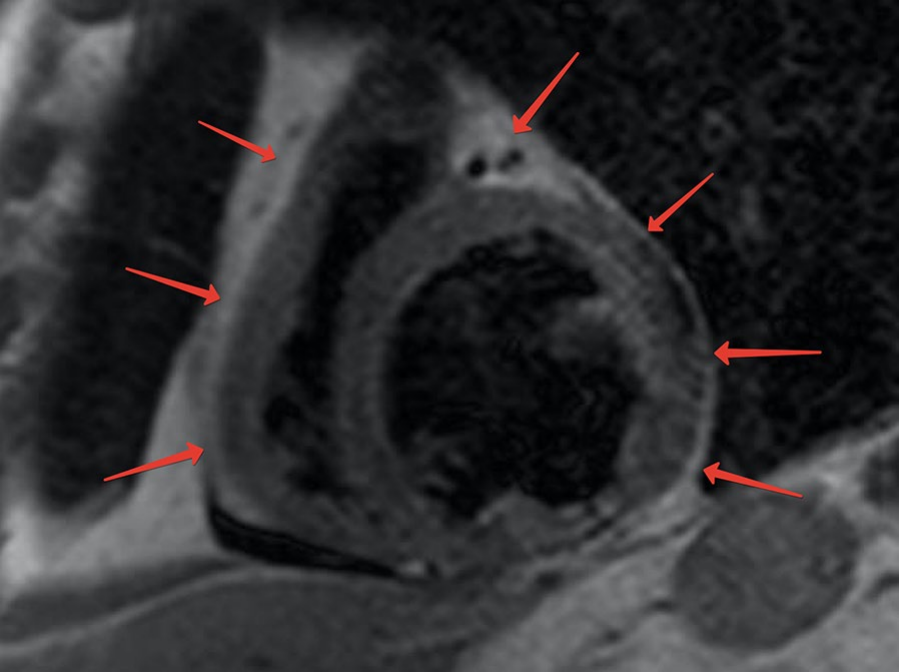
Epicardial fat (red arrows) on the MRI of the heart on a short axis

The VAT and subcutaneous adipose tissue (SAT) quantities were measured using multislice computed tomography (CT) imaging and a 64-row multidetector CT system (LightSpeed VCT-64; General Electric, Boston, MA, USA) to confirm the presence of visceral obesity, which was defined as VAT > 130 cm^2^, and a VAT to SAT ratio ≥ 0.4 [13]. The patients were assigned to two groups, and Group 1 comprised patients without visceral obesity (*n* = 29) (mean age: 56.0 [range: 51.5–63.5] years), and Group 2 comprised patients with visceral obesity (*n* = 59) (mean age: 58.50 [range: 53.0–63.0] years). Table 1 presents the patients’ clinical and demographic data (Fig. 1).

**Table 1:** Patients’ clinical and demographic data

Parameter	Patients without visceral obesity, *n* = 29	Patients with visceral obesity, *n* = 59	*p* value
Age, years, mean (range)	56.0 (51.5–63.5)	58.50 (53.0–63.0)	0.710
Male gender, *n* (%)	20 (68.9)	45 (76.2)	0.613
BMI, kg/m^2^, mean (range)	25.9 (18.3–38.4)	28.7 (171–39.1)	0.005
Abdominal obesity area, cm^2^, mean (range)	357 (253–623)	541 (381–725)	0.00
VAT, cm^2^, mean (range)	108 (64–124)	197 (145–301)	0.00
SAT, cm^2^, mean (range)	253 (159–498)	316 (201–501)	0.00
VAT/SAT, mean (range)	0.42 (0.24–0.40)	0.62 (0.60–0.72)	0.00
Documented arterial hypertension, *n* (%)	19 (65.5)	40 (67.7)	0.109
Smoking, *n* (%)	16 (55.1)	29 (49.1)	0.207
Family history of CAD, *n* (%)	1 (3.4)	2 (3.4)	0.644
Hypercholesterolemia, *n* (%)	5 (17.2)	16 (27.1)	0.02
Clinical signs and symptoms of angina before MI, *n* (%)	21 (72.4)	40 (67.7)	0.023
Chronic heart failure before MI, *n* (%)	24 (82.7)	47 (79.7)	0.02
Prior MI, *n* (%)	0	3 (5.0)	
Documented diabetes mellitus, *n* (%)	1 (3.4)	10 (16.9)	0.006
Type of acute coronary syndrome			
Q-wave MI, with ST-segment elevation, *n* (%)	2 (6.9)	3 (5.0)	0.125
Non-Q-wave MI, with ST-segment elevation, *n* (%)	27 (93.1)	56 (95.0)	0.18
Acute heart failure (Killip classification)			
Class I, *n* (%)	22 (75.8)	52 (88.1)	0.541
Class II, *n* (%)	2 (6.8)	8 (13.5)	0.035
Class III, *n* (%)	1 (3.4)	0	–
Class IV, *n* (%)	0	0	–

*BMI* body mass index, *VAT* visceral adipose tissue, *SAT* subcutaneous adipose tissue, *CAD* coronary artery disease, *MI* myocardial infarction

**Fig. 2:**
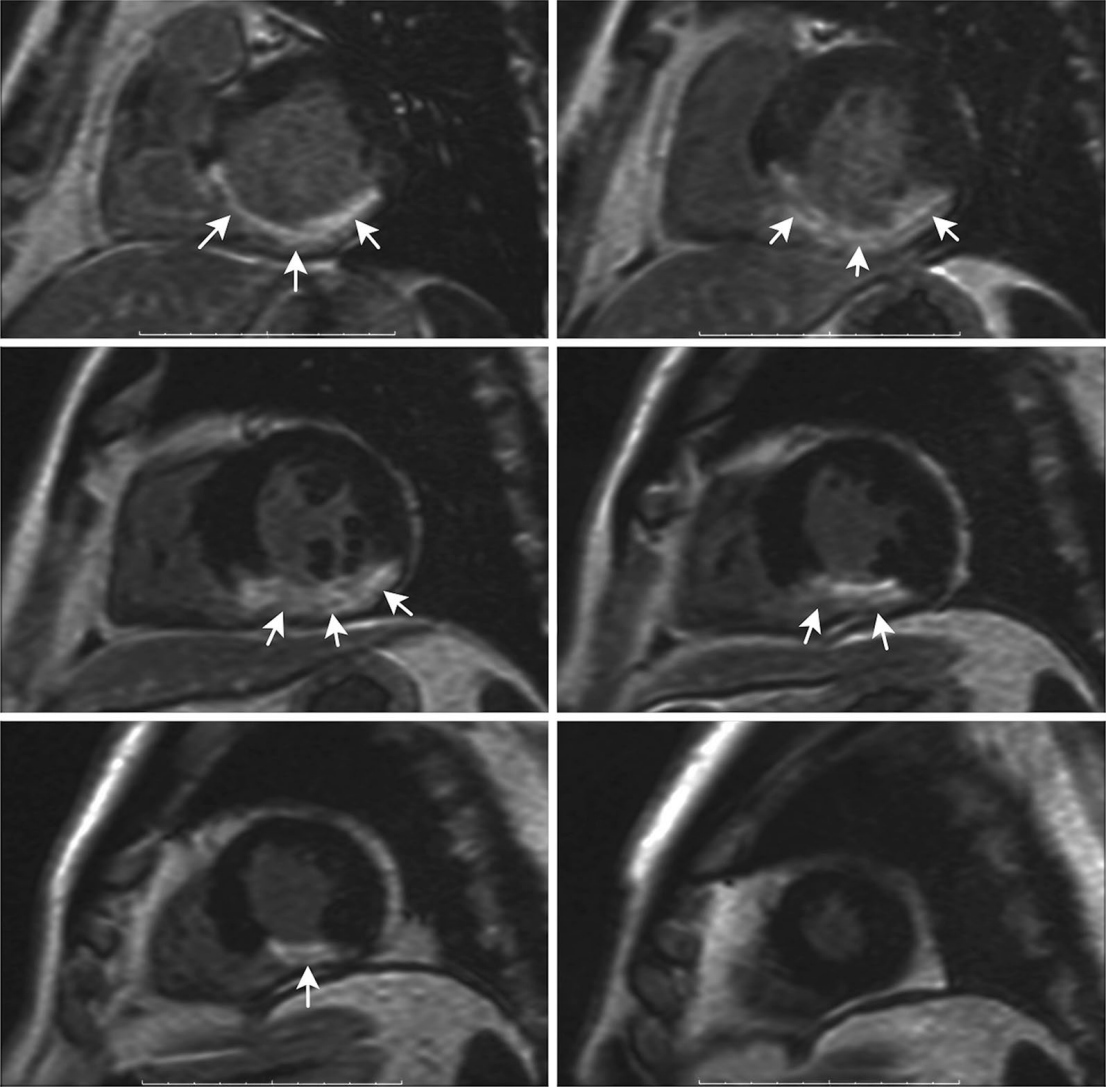
Myocardial fibrosis (white arrows) on T1-weighted images late enhancement. Short axis of the left ventricle of the heart

The EAT thickness was measured using magnetic resonance imaging (MRI) (ExcelartAtlas 1.5 MRI imager; Toshiba Corporation, Tokyo, Japan) with a magnetic field strength of 1.5 T. A T1-weighted rapid pulse sequence combined with ECG synchronization was used (echo time [TE]: 24 ms; repeat time [TR]: 1000 ms; flip angle [FA]: 90°; matrix size: 256 × 256; thickness: 7 mm). The sections were orientated to the left ventricular short axis. Using standard instruments, a layer of adipose tissue was measured at the levels of the outer edge of the myocardium to the visceral pericardial sheet along the anterior wall of the right ventricle (RV) and along the posterior wall of the left ventricle (LV) (Fig. 2). The DICOM images were processed and analyzed using Segment software, version 2.0 R 4265 (Medviso AB, Lund, Sweden).

All patients underwent contrast-enhanced MRI (ExcelartAtlas 1.5 MRI imager) with a magnetic field strength of 1.5 T at 1-year post-MI to assess myocardial scarring. Paramagnetic gadolinium medium was used (0.5 mmol/mL), and the contrast medium (0.2 mL/kg) was injected automatically into the cubital vein at 3 mL/s using a syringe injector (Optistar^®^ Elite, Tyco, Netherlands). Imaging of the cardiac fibrosis that was assessed using delayed contrast enhancement, was performed 6 min after the contrast medium was introduced using a T1-weighted pulse sequence (TE: 24 ms; TR: 1000 ms; FA: 90°; matrix: 256 × 256; thickness: 7 mm). The sections were orientated to the left ventricular short axis. The DICOM images were processed and analyzed using Segment software (Medviso). The percentage of cardiac fibrosis within the myocardium’s total mass was automatically calculated when myocardial scarring was present.

### Serum sample evaluations

The serum levels of leptin and its soluble receptor (sOB-R), adiponectin, IL-33, and ST2 were measured on days 1 and 12, and at 1-year post-MI, using commercially available enzyme-linked immunosorbent assay kits (BioVendor, Brno, Czech Republic; eBioscience, Vienna, Austria; and Critical Diagnostics, San Diego, CA, USA). Leptin sensitivity was evaluated using the free leptin index (FLI), which was defined as the ratio of the total leptin concentration in ng/mL to the sOB-R concentration in ng/mL. Leptin resistance was defined as an FLI > 0.25. The leptin to adiponectin ratio determined the presence of an adipokine imbalance.

### Statistical analysis

The statistical analyses were performed using Statistica software, version 6.1 (Dell Software, Inc., Round Rock, TX, USA) and SPSS, version 17.0 for Windows (IBM, Armonk, NY, USA). The Kolmogorov–Smirnov test was used to assess the data distributions. The data are presented as the medians and the 25th and 75th quartiles. The Mann–Whitney U test was used to compare the independent groups with skewed distributions. A Fisher’s exact test with a two-sided confidence probability was used to analyze the frequencies of the categories in two independent groups. A value of *p* < 0.05 was considered statistically significant.

## Results

### Patients’ characteristics

The study groups were comparable with respect to age, gender, and the presence of risk factors for coronary artery disease (CAD), including arterial hypertension, smoking, and a family history of CAD (*p* > 0.05). Patients with visceral obesity tended to be heavier, and to have hypercholesterolemia, angina pectoris, and type 2 diabetes mellitus. Chronic heart failure was more regularly recorded in patients without visceral obesity. The study groups did not differ significantly regarding the hospitalization duration and the therapies administered (Table 1).

The MRI findings showed a more pronounced accumulation of fat in the epicardial adipocytes in patients with visceral obesity compared to those without visceral obesity. The thicknesses of the EAT depots at the levels of the LV and RV were 1.43-times and 1.75-times higher, respectively, in patients with visceral obesity compared with those without visceral obesity (Table 2).

**Table 2:** Accumulation of fat in the epicardium according to the presence of visceral obesity

Parameter	Patients without visceral obesity, *n* = 29	Patients with visceral obesity, *n* = 59
lEAT, mm	2.8 (2.1, 6.5)	4.9 (2.6, 9.6)^а^
rEAT, mm	4.1 (3.8, 6.5)	5.9 (3.5, 10.5)^а^

lEAT, layer of the adipose tissue measured at the level of the outer edge of the myocardium to the visceral pericardial sheet along the posterior wall of the left ventricle; rEAT, layer of the adipose tissue measured at the level of the outer edge of the myocardium to the visceral pericardial sheet along the posterior wall of the right ventricle

The data presented are the medians and the 25th and 75th quartiles. ^a^*p* < 0.05 for comparisons between the study groups

### Associations between epicardial adipose tissue thickness and adipo-fibrokine indicator profiles post-myocardial infarction

**Fig. 3:**
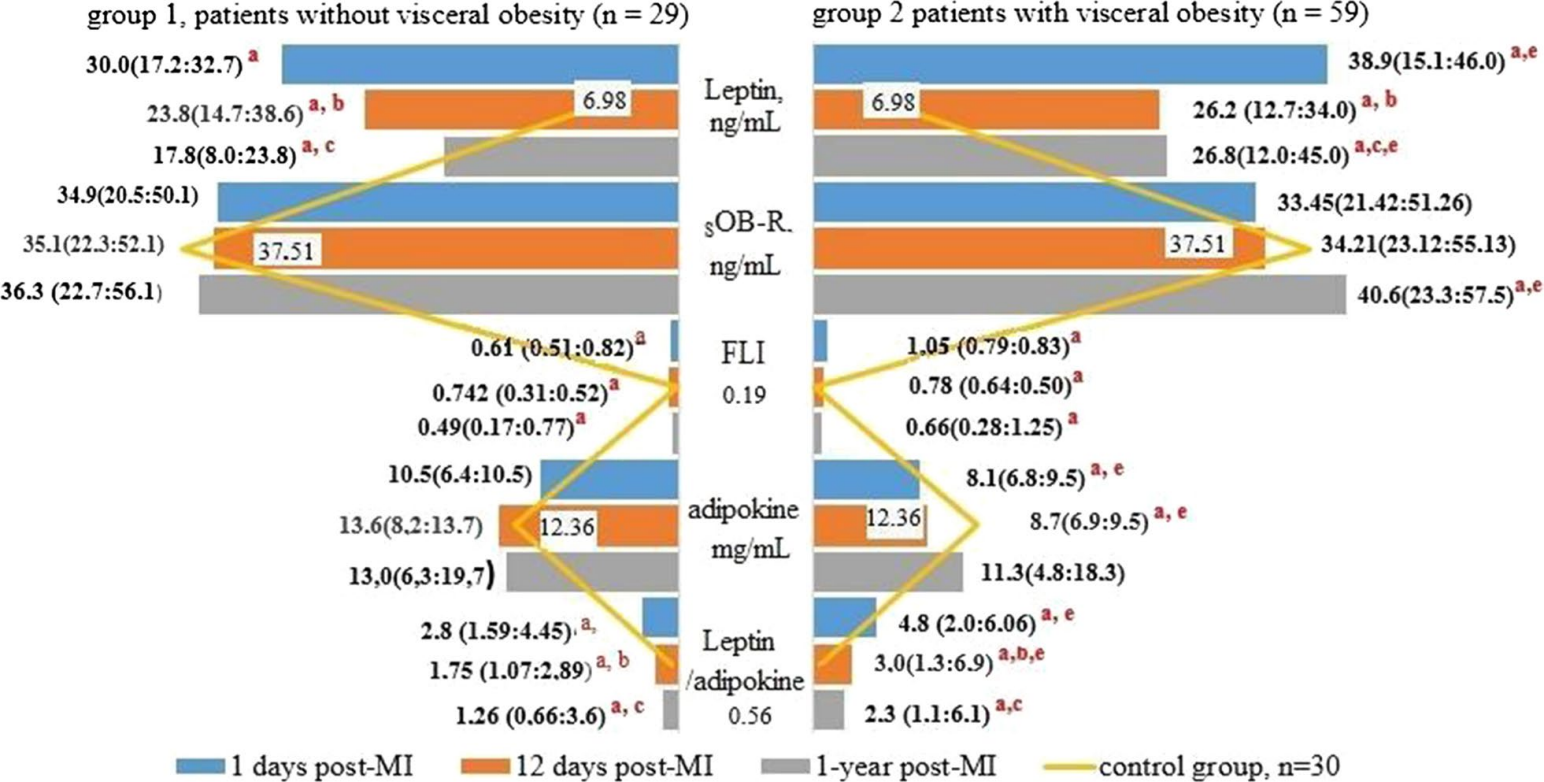
Levels of adipokines in patients with myocardial infarctions according to the presence of visceral obesity. *OB-R* soluble leptin receptor, *FLI* free leptin index, *MI* myocardial infarction. The data presented are the medians and the 25th and 75th quartiles. ^a^*p* < 0.05 compared with the control group, ^b^*p* < 0.05 for the levels measured on days 1 and 12 post-MI; ^c^*p* < 0.05 for the levels measured on day 1 and at 1-year post-MI; ^d^*p* < 0.05 for the levels measured on day 12 and at 1-year post-MI; ^e^*p* < 0.05 for comparisons between the study groups

Compared with the control individuals, the acute MI phase was accompanied by elevated leptin levels in both patient groups (Fig. 3). The leptin level changes in patients with visceral obesity were > 1.3-fold greater compared to patients without visceral obesity and fivefold greater compared to controls (*p* < 0.05). During follow-up, the leptin levels declined in both study groups, but they did not match the reference values. On day 12 post-MI, the leptin levels in patients with visceral obesity were 3.83-times higher compared to the control individuals and 2.55-times higher compared to patients without visceral obesity. There was no statistically significant difference between the study groups regarding the leptin levels (*p* > 0.05). At 1-year post-MI, the leptin levels were 1.5-times higher in patients with visceral obesity compared to patients without visceral obesity. The sOB-R levels were similar in both study groups, but at 1-year post-MI, they increased by 10% in the patients with visceral obesity compared to the control individuals and patients without visceral obesity (*p* < 0.05). The FLIs > 0.25 suggested the presence of leptin resistance in both study groups during the follow-up period. During hospitalization, the adiponectin levels declined by 35.7% in patients with visceral obesity and by 14.6% in patients without visceral obesity compared to the control individuals (*p* < 0.05). In patients without visceral obesity, the adiponectin levels increased rapidly and reached levels that were similar to those present in the control individuals on day 12 post-MI. In patients with visceral obesity, the normalization of the adiponectin levels was observed at 1-year post-MI. During the follow-up period, the leptin to adiponectin ratios in patients with visceral obesity exceeded those calculated for the control individuals and patients without visceral obesity.

Cardiac fibrosis was diagnosed in 79.1% of patients with visceral obesity and in 72.9% of patients without visceral obesity (*p* = 0.03) at 1-year post-MI. The sizes of the areas of cardiac fibrosis did not differ between patients with and without visceral obesity (10.1% vs. 12.9%). Cardiac fibrosis was absent in more of the patients without visceral obesity than those with visceral obesity (27% vs. 21%, *p* = 0.03). Cardiac fibrosis affected 1–5% of the myocardium in 33.3 and 22.7% of the patients with and without visceral obesity, respectively. More severe cardiac fibrosis affecting 20% of the myocardium was more common in patients without visceral obesity compared with those with visceral obesity (*p* = 0.03). The prevalence of cardiac fibrosis was higher in patients with visceral obesity compared to patients without visceral obesity at 1-year post-MI, and this difference was statistically significant.

Differences were found in relation to the ST2/IL-33 signaling system depending on the presence of visceral obesity. On day 1 post-MI, the ST2 levels were 3.5-times higher in patients without visceral obesity compared to controls, and they were 2.4-times higher in patients without visceral obesity compared to patients with visceral obesity (*p* < 0.05). On day 12 post-MI, the ST2 levels were 3-times lower in patients without visceral obesity and 1.6-times lower in patients with visceral obesity compared with those on day 1 post-MI (*p* < 0.05). At 1-year post-MI, the ST2 levels in the study groups did not match those in the control individuals.

The IL-33 levels in the patients without visceral obesity were 9.2-times higher compared to control individuals and 1.1-times higher compared to patients with visceral obesity (*p* < 0.05). On day 12 post-MI, a slight but statistically significant decrease in the IL-33 levels occurred in both study groups, but these levels did not match the control group’s levels. The IL-33 levels had increased by twofold in the study groups at 1-year post-MI compared with the IL-33 levels on day 1 post-MI and by 17-fold compared with the IL-33 levels in the control individuals (*p* < 0.05) (Table 3).

**Table 3:** Stimulating growth factor 2 and interleukin-33 levels in patients with myocardial infarctions according to the presence of visceral obesity

Parameter	Control group *n* = 30	Patients without visceral obesity *n* = 29			Patients with visceral obesity *n* = 59		
		Day 1	Day 12	1 year	Day 1	Day 12	1 year
ST2, ng/mL	18.81 (15.12, 21.03)	66.7 (26.2, 157.6)^a^	24.5 (17.1, 34.8)^a^	21.7 (13.9: 30.2)^a,b,с^	46.5 (21.8: 102.0)^a,е^	22.3 (16.4: 27.9)^a,е^	23.7 (16.4: 32.4)^a,b,с^
IL-33, ng/mL	0.57 (0.29, 0.69)	5.3 (3.6, 9.9)^a^	3.6 (2.8, 4.4)^a^	9.9 (8.4, 11.3)^a,b,с,d^	4.49 (4.0, 5.1)^a,е^	3.8 (3.2, 4.3)^a,е^	9.9 (9.7, 9.1: 11.3)^a,b,с,d^

*ST* stimulating growth factor, *IL* interleukin

The data presented are the medians and the 25th and 75th quartiles. ^a^*p* < 0.05 for comparisons with the control group; ^b^*p* < 0.05 for comparisons between the levels measured on days 1 and 12 post-myocardial infarction (MI); ^c^*p* < 0.05 for comparisons between the levels measured on day 1 and at 1-year post-MI; ^d^*p* < 0.05 for comparisons between the levels measured on day 12 and at 1-year post-MI; ^e^*p* < 0.05 for comparisons between the study groups

The patients with visceral obesity demonstrated more pronounced adiponectin level declines, leptin level increases, and leptin resistance during hospitalization. They were more likely to have cardiac fibrosis at 1-year post-MI compared with patients without visceral obesity. The patients without visceral obesity had higher IL-33 and ST2 levels during hospitalization, and they had more pronounced cardiosclerotic myocardial changes and a lower prevalence of cardiac fibrosis.

Correlations were found between the adipokine levels, the ST2/IL-33 signaling system, and cardiac fibrosis (Table 4). Higher adiponectin levels on day 12 post-MI were associated with less severe myocardial sclerosis in patients without visceral obesity. Likewise, higher sOB-R levels during hospitalization were associated with lower levels of fibrosis in patients without visceral obesity.

**Table 4:** Correlations between epicardial adipose tissue thickness and degree of cardiac fibrosis in patients with myocardial infarctions

Parameter	lEAT	rEAT
% fibrosis in the patients without visceral obesity	*r* = 0.34; *p* = 0.02	*r* = 0.27; *p* = 0.03
% fibrosis in the patients with visceral obesity	*r* = 0.51; *p* = 0.02	*r* = 0.51; *p* = 0.04

*r*, correlation coefficient; lEAT, layer of the adipose tissue measured at the level of the outer edge of the myocardium to the visceral pericardial sheet along the posterior wall of the left ventricle; rEAT layer of the adipose tissue measured at the level of the outer edge of the myocardium to the visceral pericardial sheet along the posterior wall of the right ventricle

Increases in the ST2 levels during hospitalization were positively associated with the degree of cardiac fibrosis at 1-year post-MI in patients with and without visceral obesity. An increase in the IL-33/ST2 ratio, indicating an excess of antifibrotic IL-33, inhibited the development of fibrosis (Table 4).

**Fig. 4:**
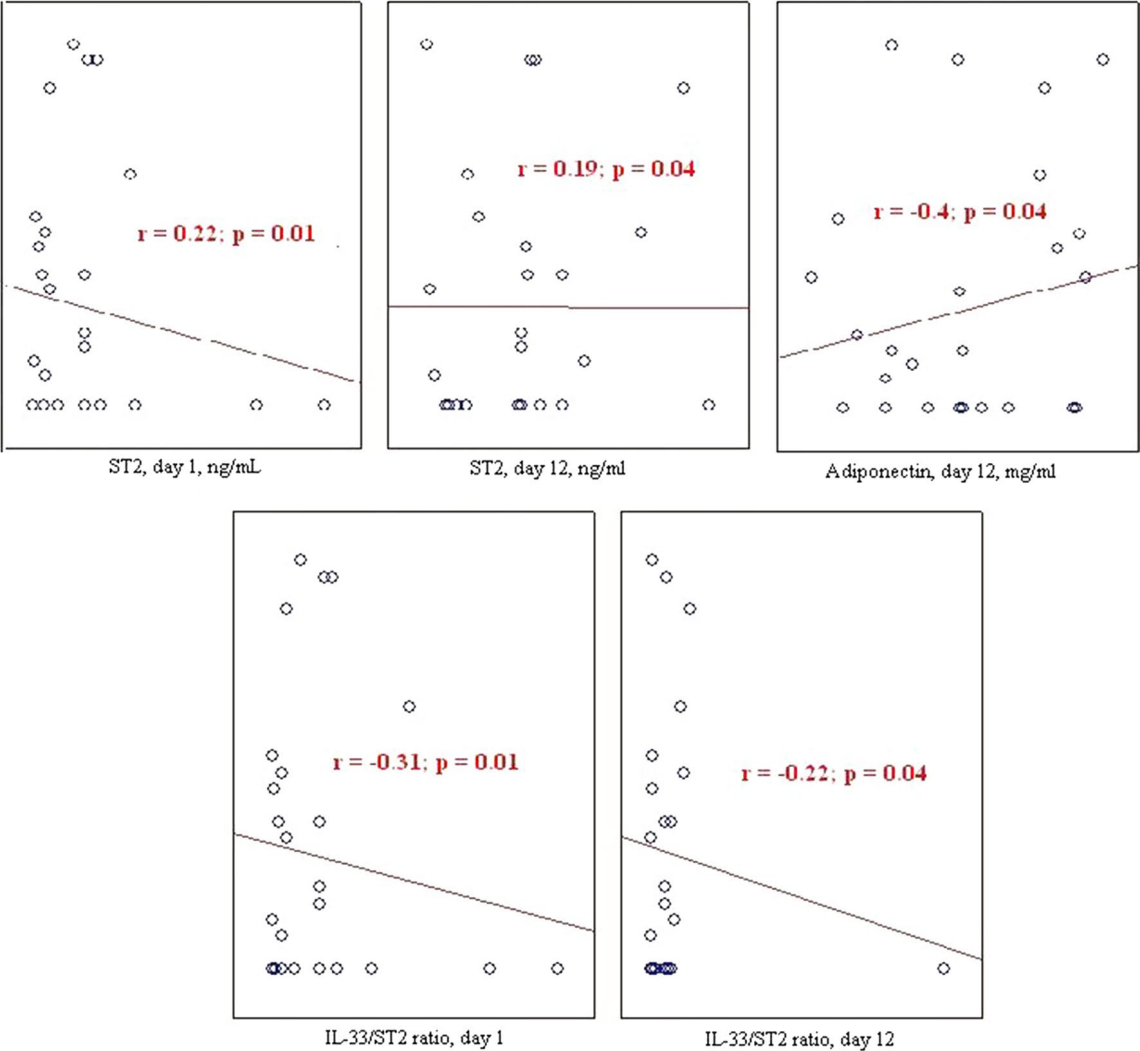
Correlations between the adipokines and the stimulating growth factor 2/interleukin-33 and the degree of cardiac fibrosis in patients without visceral obesity. *sOB-R* soluble leptin receptor, *ST* stimulating growth factor, *IL* interleukin, *MI* myocardial infarction, *r* correlation coefficient

**Fig. 5:**
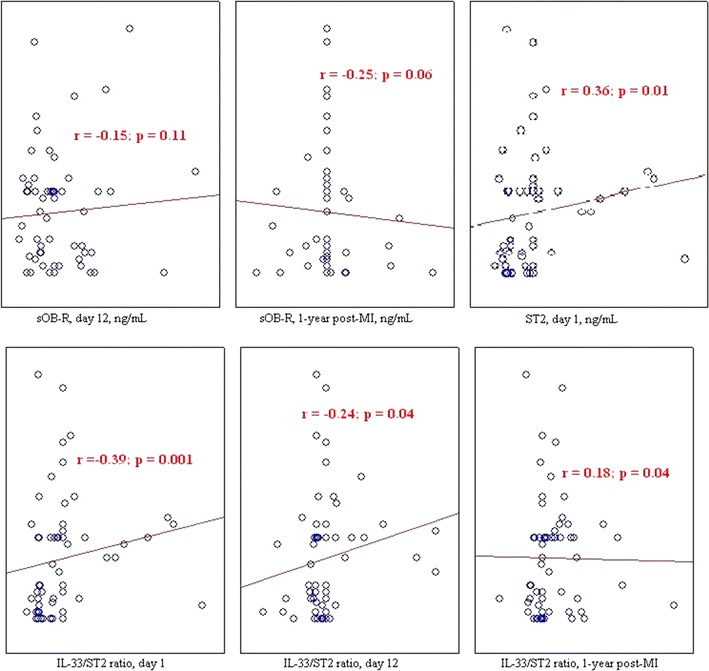
Correlations between the adipokines and the stimulating growth factor 2/interleukin-33 and the degree of cardiac fibrosis in patients with visceral obesity

The correlation analysis (Figs. 4, 5) determined that the prevalence of cardiac fibrosis in the MI patients with visceral obesity was positively correlated with the EAT thickness at the LV (*r* = 0.51, *p* = 0.02) and RV (*r* = 0.51, *p* = 0.04) levels. There was no correlation between the EAT thickness and the adiponectin levels. Negative correlations between the leptin levels at 1-year post-MI and the EAT thickness at the LV and RV levels (*r* = − 0.28, p = 0.02 and *r* = − 0.33, *p* = 0.02, respectively) were determined in the patients with visceral obesity, but not in those without visceral obesity. Negative correlations were determined between the EAT thickness and the FLIs during hospitalization (*r* = − 0.28, *p* = 0.03) and at 1-year post-MI (*r* = − 0.22, *p* = 0.04). Positive correlations between the IL-33 levels, the ST2/IL-33 signaling system, and the EAT thickness were determined in patients with and without visceral obesity during hospitalization (*r* = 0.24, *p* = 0.05 and r = 0.46, *p* = 0.04, respectively) and at 1-year post-MI (*r* = 0.33, *p* = 0.01 and *r* = 0.34, *p* = 0.02, respectively). The ST2 levels were negatively correlated with the EAT thickness (*r* = − 0.38, *p* = 0.04).

Thus, visceral obesity in patients with MI was associated with an increased EAT thickness, an adipokine imbalance, elevated leptin levels, reduced adiponectin levels during early hospitalization, and with the development of cardiac fibrosis. Higher IL-33 and ST2 levels in patients without visceral obesity during early hospitalization were followed by a lower prevalence of cardiac fibrosis after discharge. The EAT thickness was positively correlated with the degree of visceral obesity and the IL-33 levels, and negatively correlated with the ST2 levels.

## Discussion

### Relationship between leptin and adiponectin with the degree of cardiac fibrosis

EAT is a unique and multifaceted fat depot that exerts local and systemic effects [14–16], and its thickness and metabolic activity are positively associated with the severity of CAD and left ventricular myocardial thickness [17]. Since no muscle fascia separates the EAT from the myocardium, the direct paracrine effects of the adipocytokines in EAT can regulate the heart’s metabolism. We hypothesized that the adipokines in EAT may be associated with the development of cardiac fibrosis. Indeed, increased leptin levels in EAT associate with cardiac fibrosis, while increased adiponectin levels protect against cardiac fibrosis [18]. We determined that MI in patients with visceral obesity was accompanied by a significant decrease in the adiponectin level, an increase in the leptin level, and pronounced leptin resistance. The correlation analysis did not determine a statistically significant relationship between the leptin level during hospitalization and the EAT thickness in patients with visceral obesity. However, at 1-year post-MI, a negative correlation was determined between the leptin level and the EAT thickness at the LV and RV levels in patients with visceral obesity. VAT, which is located in the abdominal cavity, is thought to be the most significant source of leptin, and this would contribute to its excessive production in patients with visceral obesity and explain the discrepancy between high leptin levels and EAT thickness.

This study’s results showed a direct correlation between EAT thickness at the RV and LV levels and the degree of cardiac fibrosis. However, a key role for leptin in cardiac fibrosis was not confirmed, and there was no correlation between the leptin level and the degree of cardiac fibrosis, which may have been a consequence of the presence of leptin resistance that was confirmed by the high FLI. This notion is further supported by the negative correlation between the sOB-R level and the degree of cardiac fibrosis.

The adiponectin levels in MI patients with visceral obesity were fairly low during hospitalization, and they were similar to those in patients without visceral obesity. The leptin to adiponectin ratio can indicate an adipokine imbalance [19], and it increased because of the elevated leptin levels and the low adiponectin levels in all patients during hospitalization and at 1-year post-MI. The adiponectin levels on day 12 and the degree of fibrosis in patients without visceral obesity were negatively correlated; hence, an increase in adiponectin was associated with a smaller area of cardiac fibrosis. However, this correlation was evident in patients without visceral obesity only. In patients with visceral obesity, there was no correlation between the adiponectin level and the degree of cardiac fibrosis. Importantly, the presence of hypoadiponectinemia during hospitalization was not associated with EAT thickness. This may be related to SAT being the main source of adiponectin, which does not have a close anatomical connection with the myocardium.

### Relationship of ST2 and IL 33 with the degree of cardiac fibrosis

ST2 is one of the most promising biomarkers of cardiac fibrosis [11]. Weir et al. [20] showed that ST2 can predict functional recovery and LV remodeling during the post-infarction period. The serum ST2 level is positively correlated with the degree of coronary artery stenosis, and ST2 is a predictor of unfavorable functional outcomes and abnormal left ventricular remodeling [21]. We found that the ST2 levels were elevated during hospitalization, and that they remained elevated in all of the patients during the 1-year follow-up period. The ST2 levels were positively associated with the degree of cardiac fibrosis. Notably, the ST2 levels were higher in patients without visceral obesity during hospitalization, and the patients in this group whose ST2 level was 81.1 ng/mL often had cardiac fibrosis that comprised 20% of the myocardium. However, 33% of patients with visceral obesity had cardiac fibrosis that comprised 1–5% of the myocardium, and the ST2 level was 28.6 ng/mL in these patients. These findings indicate the presence of an association between elevated ST2 levels and the degree of cardiac fibrosis.

The correlation analysis determined the presence of an inverse relationship between the ST2 levels and EAT thickness at the RV and LV levels. There are no published data that describe the synthesis of ST2 in adipose tissue. The endothelial cells are the main source of ST2, and it is possible that the increase in the ST2 levels associated with MI is not related to EAT and that it may be a consequence of endothelial activation, which is common in MI.

ST2 mainly potentiates the effects of IL-33. During hospitalization, the IL-33 levels increased by eightfold and 9.2-fold in patients with and without visceral obesity, respectively. At 1-year post-MI, the IL-33 levels were 17-times higher in all of the patients compared with controls. IL-33 has antihypertrophic and antifibrotic effects, and an increase in the IL-33 level may protect against the development of cardiac fibrosis. Hence, the elevated IL-33 levels in MI patients without visceral obesity may have limited cardiac fibrosis compared with that in MI patients with visceral obesity. Of the MI patients without visceral obesity, 27% did not have cardiac fibrosis, and 21% of MI patients with visceral obesity did not have cardiac fibrosis. The degree of fibrosis was inversely related to the IL-33 levels and the ST2/IL-33 signaling system, which confirms the antifibrotic effects of IL-33 [21, 22]. The IL-33 level and the ST2/IL-33 signaling system were positively associated with EAT thickness at the LV and RV levels. An increase in the level of IL-33 synthesized in the endothelium and EAT thickness may be indicative of the pathogenic processes associated with CVD, namely endothelial dysfunction and lipid metabolism disorders. Increases in the IL-33 and ST2 levels are closely related to the activation of the endotheliocytes within the microcirculatory bed of the adipose tissue and the expression of leptin in adipocytes [20].

This study’s findings suggest that EAT may produce favorable levels of protective IL-33. In addition to its adverse effects, EAT serves as a local energy supplier, a buffer against excess free fatty acid levels, and as a source of protective cytokines and adipokines, including IL-10. Our data suggest that compared with patients without visceral obesity, an increase in the EAT thickness in patients with visceral obesity is accompanied by lower levels of endothelial IL-33 and ST2 during hospitalization and a smaller area of cardiac fibrosis (1–5% tissue damage). An increase in the levels of protective IL-33 cannot entirely prevent the development of cardiac fibrosis in these patients. Epicardial adipocytes can synthesize their own adipo-fibrokines, including activin A and MMP8, and Venteclef et al. [5] demonstrated their prognostic potential in the diagnosis of cardiac fibrosis, depending on the EAT thickness. In addition, IL-33 stimulates the expression of IL-6, IL-8, monocyte chemoattractant protein-1, vascular adhesion molecule-1, intercellular adhesion molecule-1, and endothelial-selectin, thereby inducing the activation of endothelial cells against the inflammatory phenotype.

In the previous study, we showed that EAT-derived and circulating secreted frizzled-related protein 4 (SFRP4) expression levels are increased in patients with CAD and in epicardial adipocytes of CAD patients, the concentrations of leptin, tumor necrosis factor-α, and IL-1, were higher, while the levels of adiponectin, IL-10, and FGF-β were lower than those in subcutaneous adipocytes [23, 24]. Bouchi et al. showed that sodium-glucose co-transporter-2 (SGLT2) inhibitors could reduce the epicardial fat volume and reduce the secretion of pro-inflammatory chemokines [25–27]. In turn, early diastolic dysfunction in individuals with obesity and type 2 diabetes correlates with BMI and epicardial fat, and EAT thickness is associated with coronary vasospasm and coronary atherosclerosis [28, 29].

## Conclusions

An increase in EAT thickness was closely related to the development of cardiac fibrosis at 1-year post-MI. The EAT was thicker in patients with visceral obesity who had more pronounced adipokine imbalances, that is, lower levels of protective adiponectin, higher leptin levels, and leptin resistance. The metabolic activity of EAT was associated with an increase in the IL-33 levels and a decrease in the ST2 levels. The degree of cardiac fibrosis was negatively related to the IL-33 levels and positively related to the ST2 levels.

## Abbreviations

CAD: coronary artery disease
CK: creatine kinase
CT: computed tomography
EAT: epicardial adipose tissue
FA: flip angle
FLI: free leptin index
LV: left ventricle
MI: myocardial infarction
RV: right ventricle
SAT: subcutaneous adipose tissue
sOB-R: soluble leptin receptor
ST2: stimulating growth factor 2
TE: echo time
TR: repeat time
VAT: visceral adipose tissue
IL: interleukin
